# Involvement of Paired Immunoglobulin-Like Receptor B in Cognitive Dysfunction Through Hippocampal-Dependent Synaptic Plasticity Impairments in Mice Subjected to Chronic Sleep Restriction

**DOI:** 10.1007/s12035-022-03127-4

**Published:** 2022-11-22

**Authors:** Xuying Li, Qian Zhai, Xingchun Gou, Minxue Quan, Yansong Li, Xiaohua Zhang, Bin Deng, Yi Tian, Qiang Wang, Lichao Hou

**Affiliations:** 1 Department of Anesthesiology, Xiang’an Hospital of Xiamen University, School of Medicine, Xiamen University, Xiamen, 361102 Fujian China; 2 Department of Anesthesiology, Affiliated Haikou Hospital, Xiangya Medical College of Central South University, Haikou, 570000 Hainan China; 3 Department of Anesthesiology & Center for Brain Science, The First Affiliated Hospital of Xi’an Jiaotong University, Xi’an, 710061 Shaanxi China; 4 Shaanxi Key Laboratory of Brain Disorders & Institute of Basic and Translational Medicine, Xi’an Medical University, Xi’an, 710021 Shaanxi China

**Keywords:** Chronic sleep restriction, Paired immunoglobulin-like receptor B, Synaptic plasticity, Cognitive function

## Abstract

Sleep loss is often associated with cognitive dysfunction. Alterations in the structure and function of synapses in the hippocampus are thought to underlie memory storage. Paired immunoglobulin-like receptor B (PirB) plays a negative role in various neurological diseases by inhibiting axon regeneration and synaptic plasticity. However, the contributions of PirB to the mechanisms underlying the changes in synaptic plasticity after sleep loss that ultimately promote deficits in cognitive function have not been well elucidated. Here, we showed that chronic sleep restriction (CSR) mice displayed cognitive impairment and synaptic deficits accompanied by upregulation of PirB expression in the hippocampus. Mechanistically, PirB caused the dysregulation of actin through the RhoA/ROCK2/LIMK1/cofilin signalling pathway, leading to abnormal structural and functional plasticity, which in turn resulted in cognitive dysfunction. PirB knockdown alleviated synaptic deficits and cognitive impairment after CSR by inhibiting the RhoA/ROCK2/LIMK1/cofilin signalling pathway. Moreover, we found that fasudil, a widely used ROCK2 inhibitor, could mimic the beneficial effect of PirB knockdown and ameliorate synaptic deficits and cognitive impairment, further demonstrating that PirB induced cognitive dysfunction after CSR via the RhoA/ROCK2/LIMK1/cofilin signalling pathway. Our study sheds new light on the role of PirB as an important mediator in modulating the dysfunction of synaptic plasticity and cognitive function via the RhoA/ROCK2/LIMK1/cofilin signalling pathway, which indicated that hippocampal PirB is a promising therapeutic target for counteracting cognitive impairment after CSR.

**Graphical Abstract:**

This illustration depicts the signalling pathway by PirB in mediating cognitive impairment and synaptic deficits in CSR mice. In the hippocampus of CSR mice, the expression level of PirB was significantly increased. In addition, CSR increases RhoA and ROCK2 levels and reduces levels of both LIMK1 and cofilin phosphorylation. PirB knockdown reverses cognitive impairment and synaptic plasticity disorders caused by CSR through the RhoA/ROCK2/LIMK1/cofilin signalling pathway

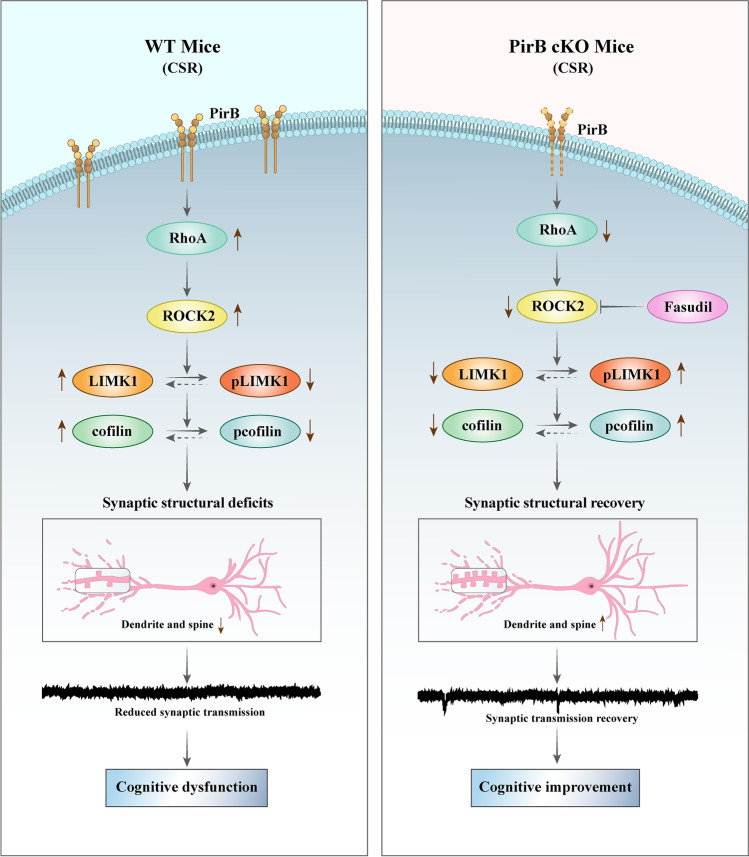

## Introduction

Sleep is a basic requirement to maintain a healthy lifestyle, with humans spending approximately 30% of their lives asleep. However, millions of people across the world are sleep-deprived because of sleep-related disorders, lifestyle choices, and so on [1, 2]. Sleep disturbances can cause a wide range of disorders throughout the body and lead to increased mortality and morbidity from cardiovascular diseases, cancer, depression, and a series of neurodegenerative diseases [3–5]. Importantly, emerging evidence has demonstrated that cognitive dysfunction is one of the most common and serious consequences induced by sleep loss. For instance, many previous studies have identified that sleep loss is a risk factor for the development of Alzheimer’s disease [6, 7]. Thus, the development of a new and effective treatment for cognitive dysfunction after sleep disturbance remains a major challenge.

Synaptic plasticity, including changes in synaptic connection strength and synaptic spine density and morphology, is important in regulating higher brain functions, such as learning and memory. Changes in synaptic connections, which are influenced by the environment throughout the lifespan, are necessary for the brain to properly promote learning and memory [8]. Notably, the synaptic plasticity of hippocampal neurons is most susceptible to being affected by sleep loss [9], and alterations in synaptic structure and functional plasticity are among the most serious effects of sleep loss and ultimately manifest as cognitive impairment [10]. A recent study showed that a brief period of 5 h of acute sleep deprivation (SD) decreases the number of dendritic spines of all subtypes of neurons in the hippocampal CA1 by 30% and leads to cognitive impairments in young adult mice [11]. Therefore, synaptic plasticity in the hippocampus may be a target for intervention to alleviate cognitive impairment after sleep loss.

Paired immunoglobulin-like receptor B (PirB), which is an orthologue of human leukocyte immunoglobulin (Ig)-like receptor B2 (LILRB2), is a type-I transmembrane glycoprotein consisting of an extracellular portion containing six Ig-like domains, a hydrophobic transmembrane segment and a cytoplasmic portion containing three immunoreceptor tyrosine-based inhibitory motifs (ITIMs) and one ITIM-like sequence [12, 13]. In addition to being expressed in macrophages, granulocytes, and B cells in the immune system, PirB is also widely expressed in neurons and astrocytes throughout the neocortex, hippocampus, cerebellum, and olfactory bulb in the central nervous system [14–16]. Previous studies have shown that PirB is highly expressed in several neurological disorders and plays a negative role by inhibiting axon regeneration and synaptic plasticity [14, 17]. Acute blockade of PirB function can enhance motor learning and performance by increasing dendritic spine stability and density in the motor cortex [18]. Moreover, germline or conditional deletion of PirB exclusively from neurons results in enhanced plasticity in the visual cortex and hippocampus associated with significantly higher spine densities [19–22]. However, little is known about whether PirB participates in CSR-induced cognitive dysfunction and, if so, which molecular mechanism underlies these changes.

Here, we uncovered a crucial role of PirB as well as its underlying mechanism in CSR-associated cognitive dysfunction. CSR upregulates PirB expression levels in the hippocampus. Knockdown of PirB improves synaptic deficits and ameliorates cognitive dysfunction. Further mechanistic analysis revealed that knockdown of PirB in the hippocampal CA1 ameliorates actin dysregulation via interaction with the RhoA/ROCK2/LIMK1/cofilin signalling pathway, leading to alterations in the structural and functional plasticity of pyramidal neurons and eventually resulting in improved cognitive dysfunction after CSR. Moreover, we identified that fasudil could mimic the beneficial effect of PirB knockdown and ameliorate synaptic deficits and cognitive impairment, which further demonstrated that the RhoA/ROCK2/LIMK1/cofilin signalling pathway is downstream of PirB in CSR. This study indicated that the PirB-RhoA/ROCK2/LIMK1/cofilin signalling pathway is a potential therapeutic target for the treatment of CSR and associated cognitive dysfunction.

## Materials and Methods

### Animals

Adult male C57BL/6 mice, aged 6–8 weeks and weighing 20–22 g, were purchased from the Laboratory Animal Center of Xi’an Jiaotong University (Xi’an, Shaanxi, China). PirB^fl/fl^ mice were obtained from the Shaanxi Key Laboratory of Brain Disorders & Institute of Basic and Translational Medicine. PirB conditional knockdown (PirB cKO) mice were established by stereotactic injection of adeno-associated virus rAAV-hSyn-Cre-WPRE-Hgh-pA (BrainVTA, Wuhan, China) into the hippocampal CA1 region of mice in the PirB^fl/fl^ mouse line. The animals were housed in groups of 5/cage in an ambient temperature (23 ± 2 °C)- and humidity (55–65%)-controlled room under a 12-h light/12-h dark cycle (light from 7:00 A.M. to 7:00 P.M.) with food and ad libitum. At the end of behavioural tests, mice were deeply anaesthetized with 2% isoflurane in oxygen. The following are indicators of successful induction of deep anesthesia: decreased respiratory rate and increased depth, loss of eyelid and corneal reflexes, reduced muscle tension and reflex responses, and no response to pain or other stimuli. After that, the mice were sacrificed by cervical dislocation. All experiments and procedures described in this study were performed in accordance with protocols approved by the Institutional Animal Care and Use Committee at Xi’an Jiaotong University (Xi’an, Shaanxi, China, No. 2019–060). Efforts were made to minimize animal suffering, and all sample sizes for the assessment parameters were calculated to minimize the number of animals used.

Number of mice per group: result 3.1 was performed from adult male C57BL/6 mice, of which 10 were used for behavioural tests, 5 for Western blot, 4 for immunofluorescence staining, and 6 for quantitative RT-PCR. Results 3.2, 3.3, and 3.5 were performed from PirB cKO mice and their wild-type littermates, of which 10 were used for behavioural tests, 5 for Western blot, 5 for immunofluorescence staining, 6 for quantitative RT-PCR, 5 for transmission electron microscopy study, and 4 for Golgi staining and Sholl’s analysis. Result 3.4 was performed from PirB cKO mice and their wild-type littermates, of which 5 were used for electrophysiology study in each group. Results 3.6 and 3.7 were performed from adult male C57BL/6 mice, of which 10 were used for behavioural tests, 5 for Western blot, 5 for immunofluorescence staining, 5 for transmission electron microscopy study, and 4 for Golgi staining and Sholl’s analysis.

### Chronic Sleep Restriction Protocols

The CSR protocols were implemented according to previously described methods [23, 24]. Briefly, CSR group mice were kept awake by being placed in slowly rotating drums (35 cm in diameter) at a constant speed (0.4 m/min) for 20 h (from 8:00 P.M. to 4:00 P.M. + 1 day), followed by 4 h (from 4:00 P.M. to 8:00 P.M.) of sleep opportunity when the drums remained stationary. CSR was repeated for 7 days. Control group mice were placed in the same plastic drums as the ones that were used for CSR for 7 days; however, the activity wheels were always stationary to permit undisturbed sleep. To reduce stress, before starting this experiment, all groups of mice were habituated to the experimental environment by being placed in the drum for 7 successive days. After that, the CSR groups of mice were exposed to the CSR experiment as described. All mice in the CSR and control groups had free access to water and food inside the drums at any time throughout the experiment. Behavioural tests were carried out immediately after CSR, and the animals were then sacrificed.

### Y Maze Test

The Y maze test was used to assess working memory and space exploratory activity as described previously [25]. Experimental mice were placed in the centre of a Y-shaped maze (arm length: 13 cm, arm width: 8 cm, height of the wall: 20 cm, Yihong Technology Co., Ltd., Wuhan, China) with three arms at 120° from each other and allowed to freely explore the three arms for 10 min. The number of arm entries and alterations were recorded automatically using Smart Video Tracking Software 3.0 (Panlab, Barcelona, Spain). The percentage of spontaneous alternation was calculated as the number of correct alterations (number of total new arm entries), which is associated with the capacity of spatial short-term memory.

### Novel Object Recognition Test (NOR)

The novel object recognition test consisted of a training phase followed by a testing phase, and since mice inherently prefer to explore novel objects, when made familiar with a specific object during the training phase, they will spend more time exploring a novel object in the test phase. Thus, a preference for the novel object indicates hippocampal memory for the familiar object. First, to reduce the levels of stress, all groups of mice were placed in the experimental room and testing box for 10 min on the 7 successive days before the training phase. After the last day of CSR, mice were trained to freely explore within a box (50 × 50 × 30 cm, Yihong Technology Co., Ltd., Wuhan, China) containing two identical items for 10 min to form object memories. Two cylindrical blocks of the same size were used as identical objects in the training test, and a conical block was used as the novel object. These objects were placed in the box at equal distances from the walls and were wiped using 10% ethanol after each test to mask any odour cues. The date of time spent with each object and the behaviour of the mice were recorded with a video camera (SNC-VB600B5, SSGE, Shanghai, China) cation placed on the old object for 10 min. The exploration time ratio for novel objects during the testing phase was analysed for each group.

### Stereotactic Injection of Virus

Six- to eight-week-old male PirB^fl/fl^ mice were anaesthetized with 2% isoflurane in oxygen. After applying eye ointment to prevent corneal drying, the mouse heads were placed on a stereotactic apparatus (RWD Life Science Co., Ltd., Shenzhen, China). The skull was exposed, and a small craniotomy was performed. Adeno-associated virus (AAV) was bilaterally microinjected into the dorsal hippocampal CA1 region using the following coordinates: anteroposterior (AP), − 2.5 mm, mediolateral (ML), ± 2 mm, dorsoventral (DV), − 1.55 mm. All skull measurements were made relative to bregma. The virus was microinjected using a 5-μL microsyringe (65,460–02, Hamilton, USA) at a flow rate of 50 nL/min controlled by a microsyringe pump. After each injection, the needle was kept in place for 10 min to allow for diffusion of the virus and was then slowly withdrawn. For PirB knockdown experiments, AAVs were injected at a volume of 1 μL/site.

### Western Blot

Western blotting was used to analyse the expression level of each target protein. In brief, the whole hippocampus of each brain was dissected and lysed in RIPA lysis buffer containing a phosphatase and protease inhibitor cocktail (Thermo Fisher Scientific, Waltham, MA, USA) for 10 min on ice. After centrifugation at 12,000 rpm for 10 min at 4 °C, the supernatant was extracted. The total protein level was measured by a bicinchoninic acid (BCA) protein assay kit (Pierce BCA Protein Assay Kit, Thermo Fisher Scientific, Waltham, MA, USA). Then, the samples were mixed thoroughly with 5 × SDS–PAGE loading buffer and RIPA lysis buffer and were boiled for 10 min at 100 °C. A total of 30 μg of protein was loaded onto an 8–15% sodium dodecyl sulphate–polyacrylamide gel and transferred onto a polyvinylidene difluoride membrane (PVDF, Millipore, Bedford, MA, USA). After blocking with 5% nonfat milk for 1–2 h at room temperature, the membranes were incubated with different primary antibodies overnight at 4 °C. The following primary antibodies were used: anti-PirB antibody (R&D Systems, AF2754, 1:1000), anti-PSD95 antibody (Santa Cruz Biotechnology, sc32290, 1:500), anti-synaptophysin antibody (Abcam, ab32127, 1:10,000), anti-RhoA antibody (Santa Cruz Biotechnology, sc418, 1:500), anti-ROCK2 antibody (Santa Cruz Biotechnology, sc398519, 1:500), anti-LIMK1 antibody (Santa Cruz Biotechnology, sc515585, 1:500), anti-pLIMK1 antibody (Thermo Fisher Scientific, PA5-104,925, 1:1000), anti-cofilin antibody (Santa Cruz Biotechnology, sc376476, 1:500), and anti-pcofilin antibody (Santa Cruz Biotechnology, sc365882, 1:500). After the membranes were washed with TBST three times, peroxidase-conjugated secondary antibodies (Diyibio, DY60202, DY60203; Beyotime, A0181, 1:5000) were used for 60 min at room temperature. The membrane bands were detected with an ECL kit (Affinity, Shanghai, China), and the densities of the protein bands were visualized and quantified with ImageJ software (NIH, MD, USA). The density of each target protein was normalized to that of its respective β-actin/alpha-tubulin. All target proteins were tested five times.

### RNA Extraction and Quantitative RT-PCR

Bilateral hippocampal tissues were harvested and shock-frozen on dry ice immediately after CSR. Total RNA was extracted using a TRIzol-based protocol. RNA extracts were then reverse transcribed by an Evo M-MLV RT Kit with gDNA Clean for qPCR II (AG11711, Accurate Biotechnology, China) at 37 °C for 15 min and 85 °C for 5 s. Reverse transcribed products were amplified by quantitative RT-PCR using an Evo M-MLV RT Kit with gDNA Clean for qPCR II (AG11711, Accurate Biotechnology, China) on a Step One Plus Real-Time PCR System, and the levels were normalized to GAPDH levels and quantified by the comparative cycle threshold method (2^−ΔΔCT^). The primer sequences were as follows: PirB forward: 5’-CCACAATGTCCATGCCACTA-3’, reverse: 5’-TTTTCCCCGATGTCTTCGTC-3’; GAPDH forward: 5’-TGTGTCCGTCGTGGATCTGA-3’, reverse: 5’-TTGCTGTTGAAGTCGCAGGAG-3’.

### Immunofluorescence Staining

Immediately after CSR, mice were anaesthetized and transcardially perfused with 0.9% saline followed by 4% paraformaldehyde (PFA). The brain tissues were postfixed with 4% PFA for 6 h at 4 °C and then dehydrated with 30% sucrose until they sank to the bottom of the container. For immunofluorescence staining, the brains were cut into 12-μm-thick cryosections using a Leica CM1950 frozen slicer. First, the cryosections were permeabilized in a solution containing 5% donkey serum albumin and 0.3% Triton for 2 h at room temperature. Then, the following primary antibodies were used separately overnight at 4 °C: anti-PirB [EPR24885-18] (Abcam, ab284407, 1:20) and anti-PSD95 (Abcam, ab238135, 1:25). After incubation with Alexa Fluor™ plus 488-donkey anti-rabbit IgG (H + L) highly cross-adsorbed secondary antibody (Invitrogen, A32790, 1:500) or Alexa Fluor™ 647-donkey anti-rabbit IgG (H + L) highly cross-adsorbed secondary antibody (Invitrogen, A31573, 1:500) for 1 h in the dark at room temperature, Alexa Fluor® 647 anti-NeuN [EPR12763] (Abcam, ab190565, 1:500) or Alexa Fluor® 594 anti-synaptophysin [YE269] (Abcam, ab206868, 1:50) was used overnight at 4 °C and the brain slices were counterstained with DAPI (Sigma, D9564, 1:500) for 5 min. All confocal images were acquired using a Leica TCS SP8 DLS microscope (Wetzlar, Germany). The number of colocalized PSD95 and synaptophysin spots in the hippocampal CA1 region was counted in 5 different fields of view for every cryosection.

### Tissue Preparation for Transmission Electron Microscopy

Transmission electron microscopy (TEM) was performed as described previously [26]. Mice were deeply anaesthetized with 2% isoflurane in oxygen and transcardially perfused with 0.9% saline followed by 4% PFA and 0.25% glutaraldehyde dissolved in 0.2 M phosphate buffer (pH 7.4). Hippocampal tissues were dissected and immersed in 2.5% glutaraldehyde in PBS and postfixed in 1% OsO4 at 4 °C. Following dehydration in a graded series of alcohols, the samples were embedded in Poly/Bed 812 resins followed by ultrathin (80 nm) sectioning with an ultramicrotome. Electron micrographs were acquired using an electron microscope (HITACHI, H7650, Japan) in the hippocampal CA1 region. The density of presynaptic vesicles was calculated by counting the number of vesicles within a defined presynaptic area, and postsynaptic PSD length, width, and synaptic cleft width were also quantified. All these evaluations were performed by using ImageJ software (NIH, MD, USA).

### Golgi Staining and Sholl’s Analysis

Golgi-Cox staining was performed as described previously [27]. Briefly, immediately after CSR, the mice were anaesthetized, and the brain tissues were freshly harvested on ice, washed with 0.9% saline, and immersed in Golgi-Cox solution (A: 5% potassium dichromate; B: 5% mercuric chloride; C: 5% potassium chromate) for 5 days in the dark at room temperature for fixation. After that, the brain tissues were transferred into a fresh Golgi-Cox solution for another 14 days and into 30% sucrose for 2 days. A vibratome (Leica, Wetzlar, Germany) was used to collect coronal Sects. (150 μm). The staining was performed using the following procedures: rinsing with deionized water (1 min), incubating with 50% NH_4_OH (30 min), placing in fixing solution (30 min), and subsequently incubating with 5% sodium thiosulfate. After washing with deionized water, the brain sections were dehydrated and cleared and then covered with a coverslip. The images were captured under the bright field of confocal microscopy (Leica TCS SP8 STED 3X, Wetzlar, Germany). The CA1 pyramidal neuron parameters, including dendritic length, branching complexity, and number of dendrites, were quantified by Sholl’s analysis using the NeuronJ plugin in ImageJ software. Imaris software (v.9.7, Bitplane, Zurich, Switzerland) was used to reconstruct and classify the synaptic spines (stubby, mushroom, long thin, and filopodia). The spine density and classification were analysed and calculated in the Imaris spine classification module.

### Electrophysiology

Slice preparation was performed as described previously [28]. Briefly, immediately after CSR, mice were deeply anaesthetized with 2% isoflurane in oxygen and transcardially perfused with ice-cold oxygenated (95% O_2_, 5% CO_2_) cutting solution containing 95 mM NaCl, 1.8 mM KCl, 1.2 mM KH_2_PO_4_, 0.5 mM CaCl_2_, 7 mM MgSO_4_, 26 mM NaHCO_3_, 15 mM glucose, and 50 mM sucrose (pH 7.4). The brain tissues were rapidly harvested and immersed in a cutting solution. A VT1200S Vibratome (Leica, Wetzlar, Germany) was used to collect hippocampal slices of 300 µm thickness. After being stored in a recording solution for 30 min at 32 ± 1 °C, the hippocampal slices were left at room temperature for 1 h for recording. The components of the recording solution were identical to those of the cutting solution except for the following: 127 mM NaCl, 2.4 mM CaCl_2_, 1.3 mM MgSO_4_, and 0 mM sucrose. Recording electrodes were placed in the fluorescence-labelled pyramidal neurons in layers II and III of the hippocampal CA1 region, which were filled with pipette solution containing 125 mM K-gluconate, 5 mM KCl, 10 mM HEPES, 0.2 mM EGTA, 1 mM MgCl_2_, 4 mM Mg-ATP, 0.3 mM Na-GTP, and 10 mM phosphocreatine (pH 7.35, 290 mOsm). Whole-cell patch-clamp recordings were made using a MultiClamp 700B amplifier (Molecular Devices, USA) and 1550A digital converter (Molecular Devices, USA). Series resistance was monitored throughout the experiments, and cells included in the analysis had resistance values of < 20 MΩ. Neurons were rejected if their membrane potentials were more positive than − 60 mV, if the ratio of Rin to Rs was < 5, or if series resistance fluctuated > 20% of initial values. Data were filtered at 3 kHz and sampled at 10 kHz. The extracellular fluid for miniature excitatory postsynaptic current (mEPSC) recording included 100 µM picrotoxin and 1 µM TTX. Before mEPSC recording, the TTX was incubated in the culture for 10 min, followed by another 10 min lead-in to establish a baseline. The voltage clamp recordings were performed at a holding potential of − 70 mV. The frequency and amplitude of mEPSCs were analysed by Mini60 (MiniAnalysis, Synaptosoft, Leonia, USA) and visually confirmed. To obtain a high signal-to-noise ratio and accurately determine the mEPSC amplitude, only events > 10 pA in mEPSCs were accepted for analysis [29].

### Administration of Fasudil

Fasudil (FAS, HA-1077, Selleck Chemicals Inc. Houston, USA) was dissolved in saline (1 μg/µL). To investigate the therapeutic effect of fasudil on CSR, fasudil and fasudil + CSR mice were intraperitoneally injected with fasudil (10 mg/kg) once daily for 14 successive days, 7 days before the CSR protocol. Control mice and CSR mice were intraperitoneally injected with an equivalent amount of vehicle solvent. All injections were carried out at 3:00 P.M. to avoid any effects due to changes in pharmacokinetics and were continued until the last day of CSR. After the injection, the control and fasudil mice were returned to their cages in another room, and the CSR and fasudil + CSR mice were maintained in the CSR box. Immediately after CSR, behavioural tests were evaluated, and the brain tissue was harvested for further tests.

### Statistical Analysis

All data are presented as the mean ± SD across the groups and were statistically analysed using GraphPad Prism 7.0 software (GraphPad Company, San Diego, CA, USA). Continuous data were tested for normal distribution and analysed by one-way ANOVA (followed by Tukey’s multiple comparisons tests) or Kruskal–Wallis test (followed by Dunn’s multiple comparisons tests). Two-way ANOVA was applied to analyse the neural dendritic complexity. A *P* value of less than 0.05 was considered statistically significant.

## Results

### CSR Induces Cognitive Impairment and Synaptic Deficits and Increases PirB Expression Levels in the Hippocampus

**Fig. 1:**
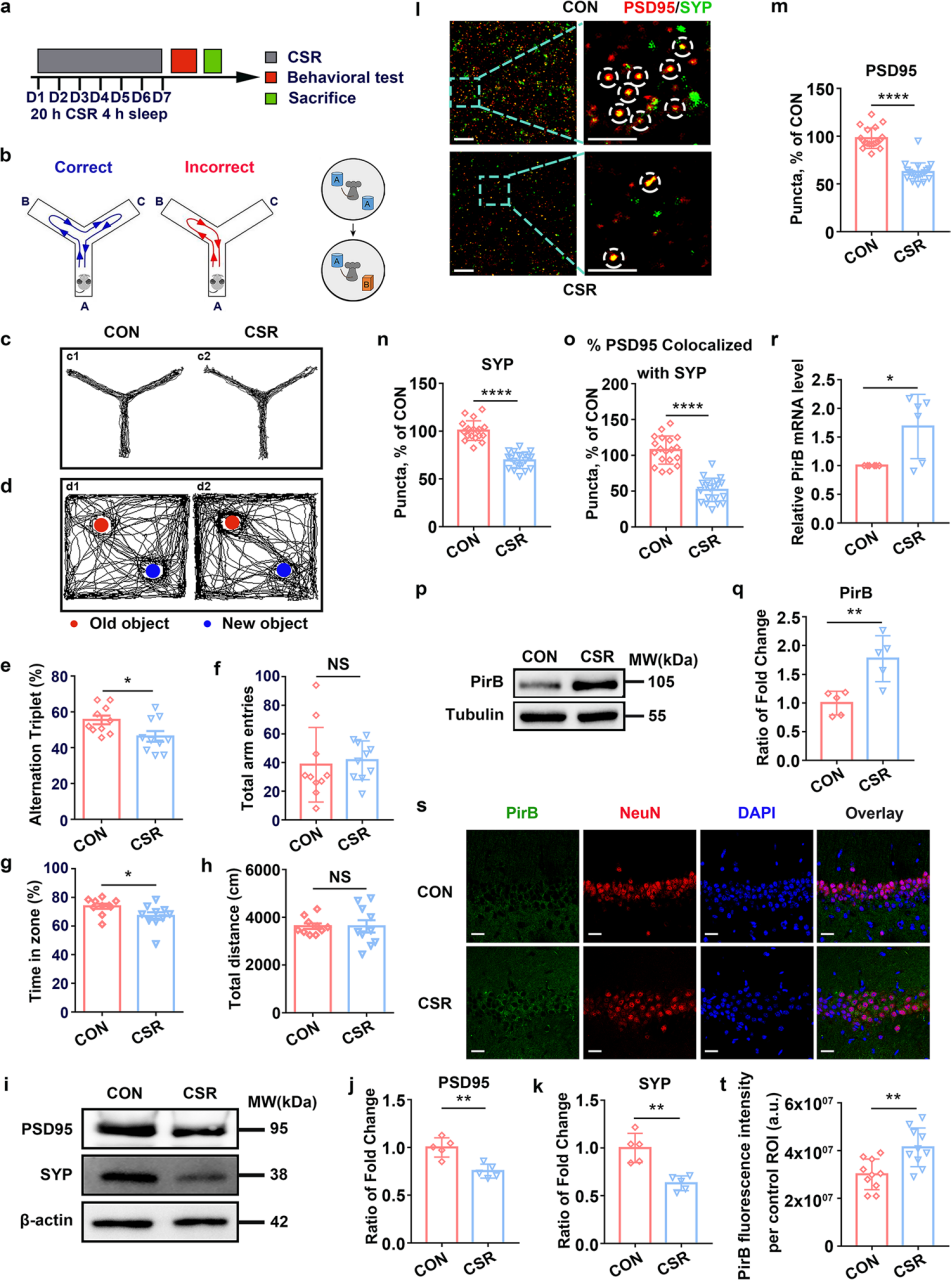
Cognitive function and the expression of synaptic proteins and PirB in the hippocampus of CSR mice. **a** The experimental protocol for CSR is presented. **b** left, **c** Representative Y maze test trajectories of control and CSR mice. **b** right, **d** Representative NOR test trajectories of control and CSR mice. **e**, **f** The percent of spontaneous alteration rates and total number of arm entries in the Y maze test were measured (*n* = 10). **g**, **h** The recognition index and total travel distance in the NOR test were measured (*n* = 10). **i** Western blot analysis of hippocampal PSD95 and SYP levels in control and CSR mice. **j**, **k** Ratio of fold change from Western blot. The PSD95 (*n* = 5) and SYP (*n* = 5) levels were significantly lower in CSR mice than in control mice. **l**–**o** Superresolution SIM images and quantification of PSD95 (red, *n* = 19–20) and SYP (green, *n* = 19–20) immunoreactive puncta and their apposition (*n* = 19–20) using Imaris in the hippocampal CA1 region of the two groups of mice. Scale bars: 5 μm. **p** Western blot analysis of hippocampal PirB levels in control and CSR mice. **q** Ratio of fold change from Western blot. The PirB level was significantly higher in CSR mice than in control mice (*n* = 5). **r** PirB was upregulated in the hippocampus at the mRNA level after CSR (*n* = 6). **s**, **t** Representative immunofluorescence images and quantification of PirB (green, *n* = 10) fluorescence intensity of the two groups of mice. Scale bars: 20 μm. Asterisks indicate significant differences between groups (2-tailed, unpaired *t* test, *p < 0.05, **p < 0.01, ****p < 0.0001). Data are presented as the means ± SD

After 7 days of CSR (Fig. 1a), mice showed significantly lower spontaneous alternation rates than those of control mice in the Y maze test (Fig. 1b left, c, e). In the novel object recognition test, CSR mice spent less time exploring a novel object during the test phase (Fig. 1b right, d, g), suggesting the deterioration of hippocampal learning and memory function. Moreover, we observed no significant differences in the total number of arm entries in the Y maze test or the total distance travelled between CSR and control mice in the novel object recognition test (Fig. 1f, h).

Presynaptic protein markers (synaptophysin, SYP) and postsynaptic protein markers (postsynaptic density protein, PSD95) play important roles in cognitive function and synaptic plasticity [30, 31]. Western blot analysis showed a dramatic decrease in the protein expression levels of both SYP and PSD95 in the hippocampus of CSR mice (Fig. 1i–k). Moreover, the colocalization of pre- and postsynaptic markers represents the structural integrity of synapses, and quantification of colocalized SYP and PSD95 puncta revealed a significant loss of synapses in CSR mice (Fig. 1l–o). These findings collectively suggested that CSR induces cognitive impairment and synaptic deficits.

To investigate whether the PirB receptor is involved in the pathological process of CSR and associated cognitive impairment, we detected the protein and mRNA levels of PirB by Western blotting, immunofluorescence staining, and qRT-PCR, and found that PirB expression in the hippocampus was significantly increased in CSR mice (Fig. 1p–t). These results may indicate a potential link between the PirB receptor and cognitive impairment as well as synaptic plasticity disorder after CSR.

### Knockdown of PirB in the Hippocampal CA1 Reverses Cognitive Impairment and Synapse Loss in CSR Mice

**Fig. 2:**
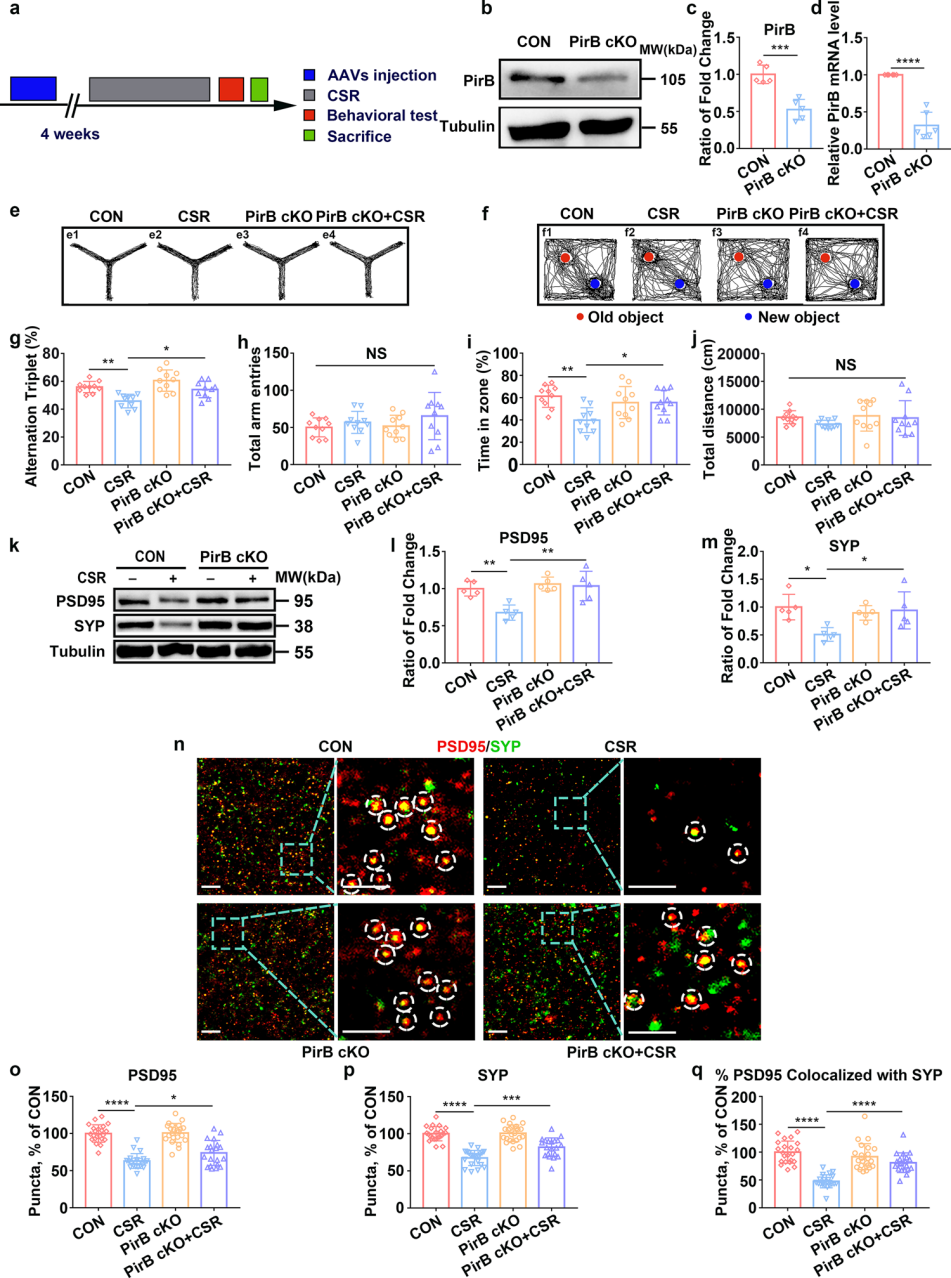
Effect of knockdown of PirB in the hippocampal CA1 region on cognitive impairment and synapse loss in CSR mice. **a** The experimental protocol for the establishment of PirB conditional knockout mice and CSR mice is presented. **b**–**d** Western blots (*n* = 5) and qRT-PCR results (*n* = 6) showing the efficiency of PirB knockdown in the hippocampus. **e**, **f** Representative Y maze and NOR test trajectories. **g**, **h** The percent of spontaneous alternation rates and total number of arm entries in the Y maze test were measured (*n* = 10). **i**, **j** The recognition index and total travel distance in the NOR test were measured (*n* = 10). **k** Western blot analysis of hippocampal PSD95 and SYP levels in the four groups of mice. **l**, **m** Ratio of fold change from Western blot. The PSD95 (*n* = 5) and SYP (*n* = 5) levels were significantly higher in PirB cKO + CSR mice than in CSR mice. **n** Superresolution SIM images of PSD95 (red) and SYP (green) immunoreactive puncta in the hippocampal CA1 region of four groups of mice. Scale bars: 5 μm. **o**–**q** Quantification of synaptic puncta and their apposition indicates significant increases in PSD95 (*n* = 20–23) and SYP (*n* = 20–23) and their apposition (*n* = 20–23) in the hippocampal CA1 region of PirB cKO + CSR mice compared to that of CSR mice. Data from **c** and **d** are presented as the means ± SD and were analysed by 2-tailed, unpaired *t* test. Data from **g**–**q** are presented as the means ± SD and were analysed by one-way ANOVA with Tukey’s test (*p < 0.05, **p < 0.01, ***p < 0.001, ****p < 0.0001)

To address the question of whether there is a causal relationship between the upregulation of PirB expression and cognitive impairment as well as synaptic plasticity disorder after CSR, we generated PirB cKO mice by stereotactically injecting adeno-associated virus (rAAV-hSyn-Cre-WPRE-Hgh-pA) into the hippocampal CA1 region of mice in the PirB^fl/fl^ mouse line (Fig. 2a). The efficiency of PirB knockdown was verified by Western blotting and qRT-PCR. The results showed that PirB knockdown resulted in 48% downregulation of PirB protein levels (*p* < 0.001), and 68% downregulation of PirB mRNA expression (*p* < 0.0001) compared with those of the control group (Fig. 2b–d). In the Y maze test, CSR mice showed significantly lower spontaneous alternation rates than those of control mice, which is consistent with previous results (Fig. 2e, g). However, PirB knockdown partially reversed the cognitive deficits after CSR (Fig. 2e, g). PirB knockdown also led to a significantly longer time taken to explore a novel object during the test phase in mice subjected to CSR than in CSR mice in the novel object recognition test (Fig. 2f, i). Moreover, no significant differences in the total number of arm entries in the Y maze test or the total distance travelled in the novel object recognition test were observed (Fig. 2h, j).

How PirB in the hippocampal CA1 region modulates the cognitive dysfunction associated with CSR remains unknown. Previous studies have suggested that structural and functional plasticity in the hippocampal CA1 region is the neurobiological basis of cognitive function, and PirB is involved in the regulation of synaptic plasticity in a variety of neuropsychiatric diseases [12, 15]. Thus, we assessed the expression of SYP and PSD95 and the synaptic density when PirB was knocked down following CSR. Western blot analysis showed dramatically higher protein expression levels of both SYP and PSD95 in the hippocampi of PirB cKO + CSR mice than in the hippocampi of CSR mice (Fig. 2k–m). Quantification of colocalized SYP and PSD95 puncta revealed a significant restoration of synapse density in PirB cKO + CSR mice (Fig. 2n–q). Together, these findings collectively suggested that PirB knockdown in the hippocampal CA1 reverses cognitive impairment and alleviates synapse loss after CSR.

### Knockdown of PirB in the Hippocampal CA1 Alleviates Synaptic Structural Deficits in CSR Mice

**Fig. 3:**
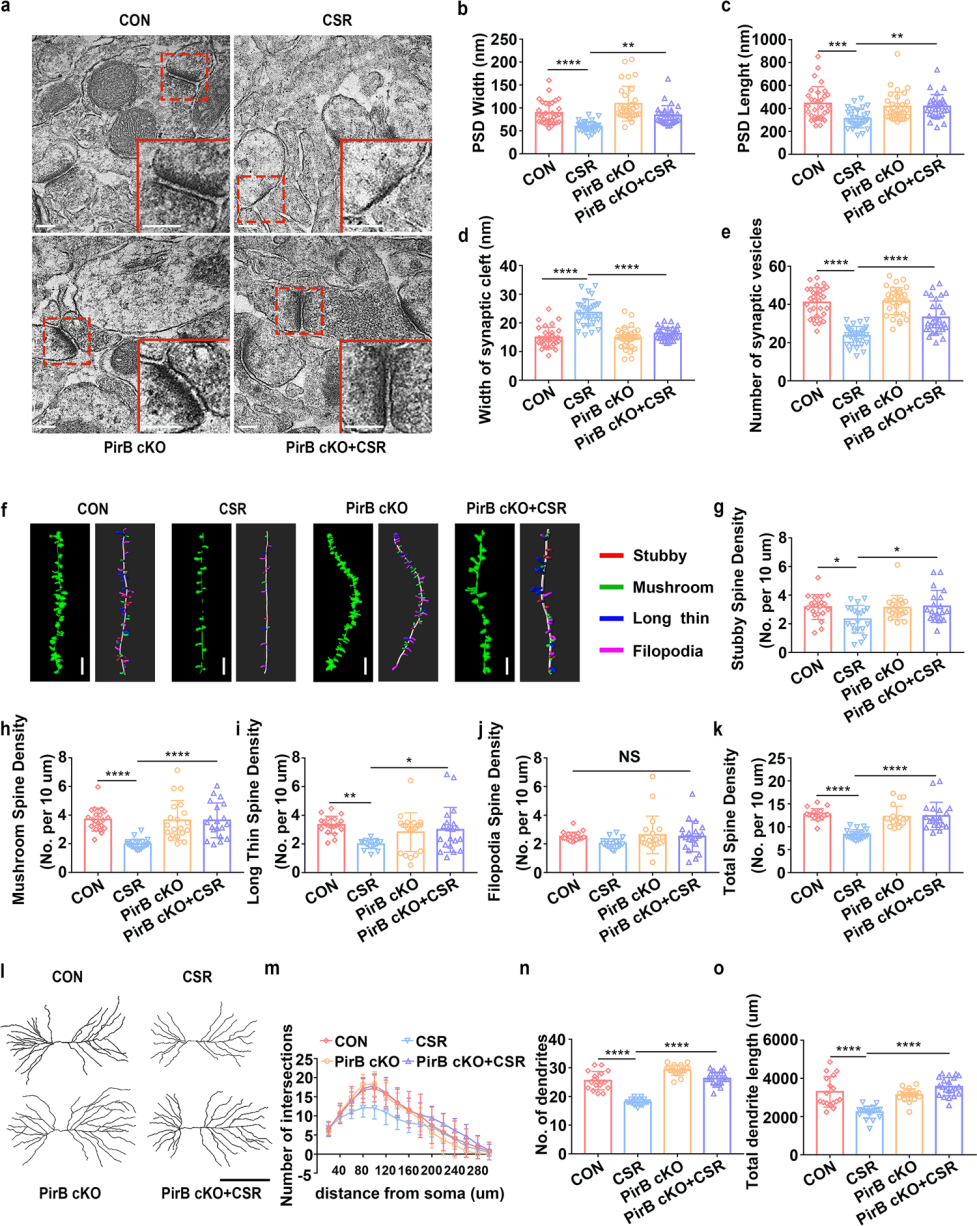
Effect of knockdown of PirB in the hippocampal CA1 region on synaptic structural deficits in CSR mice. **a** TEM study of ultrastructural features of synapses in the hippocampal CA1 region in control and CSR mice in the presence or absence of PirB. Scale bars: 200 nm. **b** The width of the PSD (*n* = 30–31). **c** The length of the PSD (*n* = 30–31). **d** The width of the synaptic cleft (*n* = 30–31). **e** The number of synaptic vesicles (*n* = 30–31). **f** Representative confocal stack and 3D reconstruction images of dendrites of hippocampal CA1 pyramidal neurons obtained from four groups of mice. Scale bars: 5 μm. **g**–**k** Summary of the density of stubby (*n* = 20)-, mushroom (*n* = 20)-, long thin (*n* = 20)-, filopodia-shaped spines (*n* = 20), and the total spine density (*n* = 20) on dendrites of hippocampal CA1 pyramidal neurons of four groups of mice. **l** Representative images of pyramidal neurons in the hippocampal CA1 region derived from the four groups of mice. Scale bars: 100 μm. **m** Sholl’s analysis of the dendritic branching complexity of the four groups of mice (*n* = 17–21). **n**, **o** Sholl’s analysis of total dendritic length and number of dendrites of four groups of mice (*n* = 17–21). Data from **b**–**e**, **g**–**k**, **n**, and **o** are presented as the means ± SD and were analysed by one-way ANOVA with Tukey’s test. Data from **m** are presented as the means ± SD and were analysed by two-way ANOVA (*p < 0.05, **p < 0.01, ***p < 0.001, ****p < 0.0001)

Next, we further assessed the effect of PirB knockdown on the structural plasticity of hippocampal CA1 neurons after CSR. The number of presynaptic vesicles and postsynaptic PSD length and width were reduced, and the width of the synaptic cleft was increased in CSR mice (Fig. 3a–e). However, these changes in synaptic ultrastructure were reversed by PirB knockdown (Fig. 3a–e), which is consistent with the immunofluorescence results showing that colocalized SYP and PSD95 puncta were restored in PirB cKO + CSR mice. Dendritic arborization and spines of hippocampal CA1 pyramidal neurons were evaluated using Golgi staining immediately after CSR. CSR mice had significantly lower proportions of stubby, mushroom, and long thin spines and lower total spine densities than those of control mice, with no obvious differences in filopodia spines, and these effects were reversed by PirB knockdown (Fig. 3f–k). PirB knockdown did not lead to higher proportions of stubby, mushroom, and long thin spines in CA1 pyramidal neurons in PirB cKO mice than in control mice without PirB knockdown, possibly due to the ceiling effect (Fig. 3f–k). Moreover, Sholl’s analysis showed that the dendritic complexity of CA1 pyramidal neurons was markedly decreased in CSR mice, which was partly reversed by PirB knockdown (Fig. 3l, m). In particular, significant differences were detected at Sholl’s radii of 60–180, 220, and 260 μm in the basal hemisphere between PirB cKO + CSR and CSR mice (Fig. 3l, m). In parallel, the total dendritic length and number of dendrites in CA1 pyramidal neurons were counted. The levels of all these parameters were significantly decreased in the CSR mice and were also rescued by PirB knockdown to levels similar to those of control mice (Fig. 3n, o). These findings suggested that the knockdown of PirB ameliorates synaptic structural deficits after CSR by increasing dendritic complexity and synaptic spine density, thereby improving cognitive function.

### Knockdown of PirB Increases Synaptic Transmission in the Hippocampal CA1

**Fig. 4:**
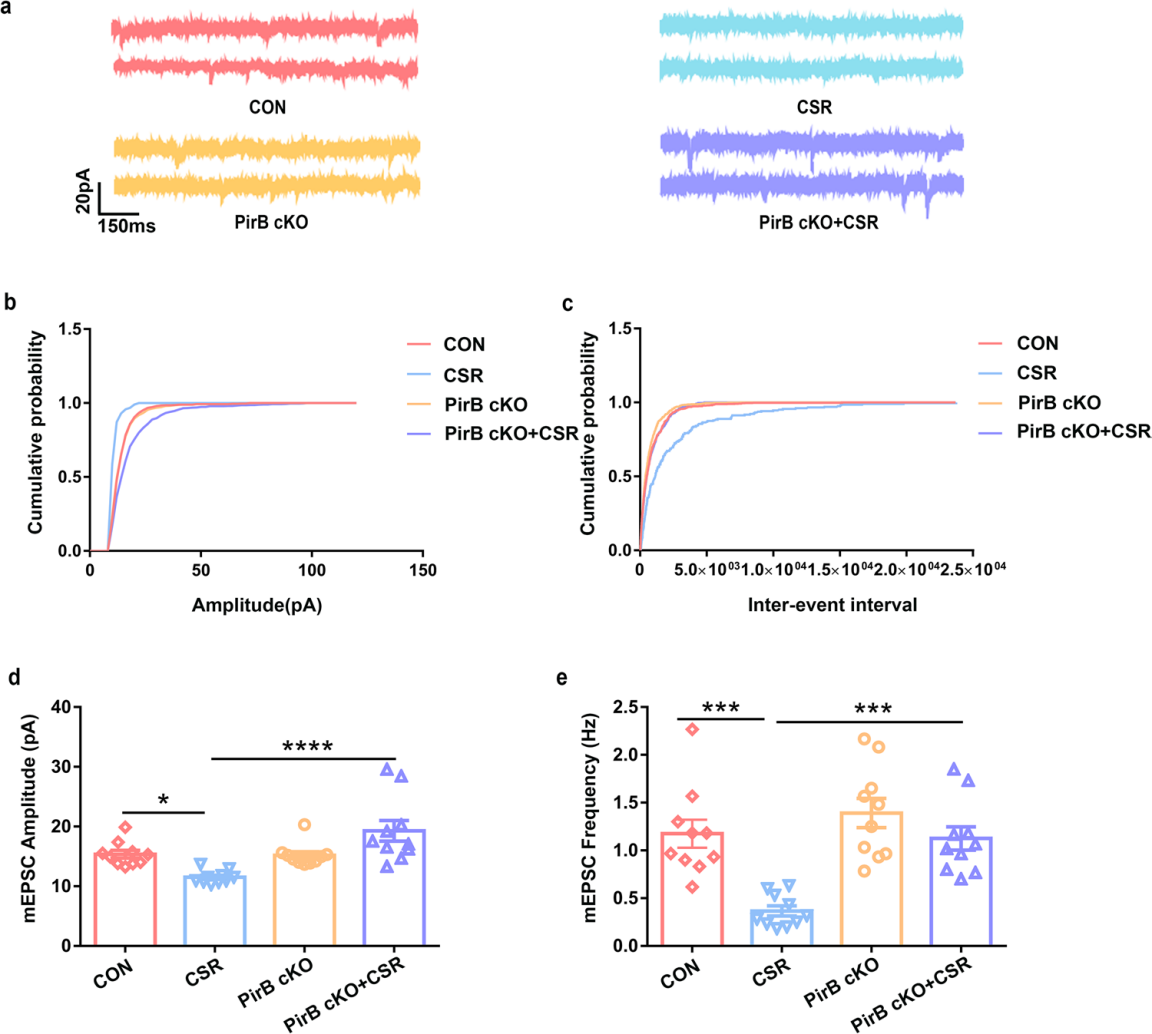
Effect of PirB knockdown on synaptic transmission in the hippocampal CA1 region. **a** Representative voltage clamp traces show mEPSCs recorded from hippocampal CA1 excitatory pyramidal neurons in slices from four groups of mice at a holding potential of − 70 mV (red: CON, blue: CSR, yellow: PirB cKO; purple: PirB cKO + CSR). **b** Cumulative probability representing the amplitude of mEPSCs in hippocampal CA1 excitatory pyramidal neurons from four groups of mice. **c** Cumulative probability represents the frequency of mEPSCs in hippocampal CA1 slices from four groups of mice. **d** Amplitudes of mEPSCs recorded in slices from the CON mice (red: *n* = 10 neurons from 5 mice), CSR mice (blue: *n* = 10 neurons from 5 mice), PirB cKO mice (yellow: *n* = 10 neurons from 5 mice), and PirB cKO + CSR mice (purple: *n* = 10 neurons from 5 mice). **e** Frequency of mEPSCs recorded in slices from the CON mice (red: *n* = 10 neurons from 5 mice), CSR mice (blue: *n* = 10 neurons from 5 mice), PirB cKO mice (yellow: *n* = 10 neurons from 5 mice), and PirB cKO + CSR mice (purple: *n* = 10 neurons from 5 mice). Data are presented as the means ± SD and were analysed by one-way ANOVA with Tukey’s test (*p < 0.05, ***p < 0.001, ****p < 0.0001)

To investigate whether excitatory synapse function is changed as a result of PirB knockdown, we performed whole-cell recordings of hippocampal CA1 pyramidal neurons to record miniature excitatory postsynaptic currents (mEPSCs) (Fig. 4a). Significant decreases in the frequency and amplitude of mEPSCs were detected in hippocampal CA1 pyramidal neurons after CSR (Fig. 4b–e). Moreover, the mEPSCs recordings from PirB cKO + CSR mice hippocampal CA1 pyramidal neurons showed significantly higher frequencies and amplitudes of mEPSCs than those of the mEPSCs of CSR mice and had levels comparable to those in control mice (Fig. 4b–e). The increase in the frequency and amplitude of mEPSCs is consistent with our synaptic molecular and morphological results, indicating that PirB knockdown in the hippocampal CA1 reversed the reduction in synaptic transmission after CSR.

### PirB Knockdown Mice Exhibit Altered RhoA/ROCK2/LIMK1/cofilin Signalling After CSR

**Fig. 5:**
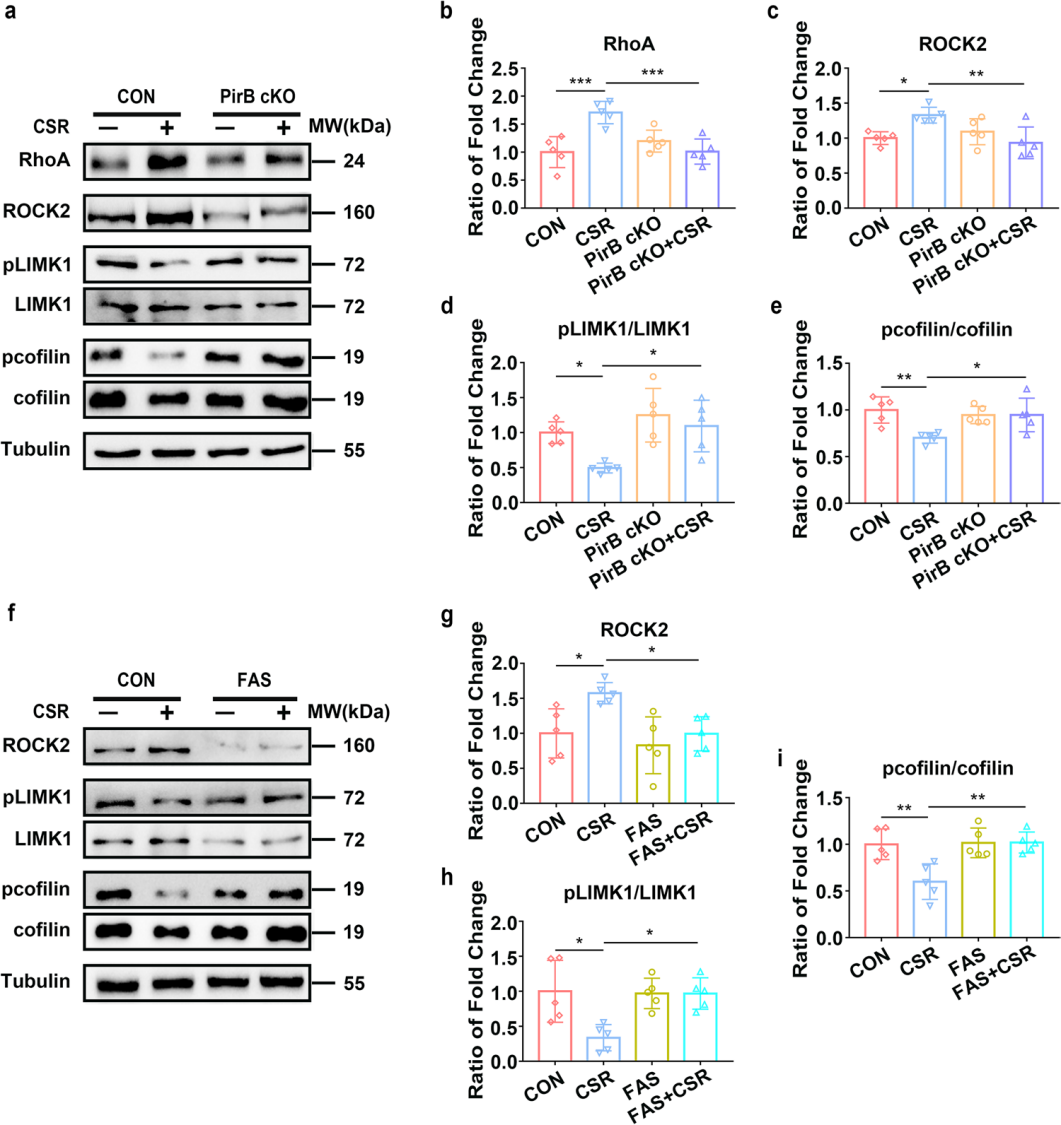
PirB activates the RhoA/ROCK2/LIMK1/cofilin signalling pathway in CSR mice. **a** Western blot analysis of hippocampal RhoA, ROCK2, pLIMK1/LIMK1, and pcofilin/cofilin levels in control, CSR, PirB cKO, and PirB cKO + CSR mice. **b**–**e** Ratio of fold change from Western blot. The RhoA (*n* = 5) and ROCK2 (*n* = 5) levels were significantly higher in CSR mice than in control and PirB cKO + CSR mice. The phosphorylated LIMK1 (*n* = 5) and cofilin (*n* = 5) levels were significantly lower in CSR mice than in control and PirB cKO + CSR mice. **f** Western blot analysis of hippocampal ROCK2, pLIMK1/LIMK1, and pcofilin/cofilin levels in control and CSR mice after fasudil treatment. **g**–**i** Ratio of fold change from Western blot. The ROCK2 level was significantly lower in fasudil + CSR mice than in CSR mice (*n* = 5). The phosphorylated LIMK1 (*n* = 5) and cofilin (*n* = 5) levels were significantly higher in fasudil + CSR mice than in CSR mice. Data are presented as the means ± SD and were analysed by one-way ANOVA with Tukey’s test (*p < 0.05, **p < 0.01)

We then investigated by what mechanism PirB regulates the synaptic deficits of hippocampal neurons and further influences cognitive function after CSR. Synaptic plasticity changes in morphology and function could be induced by the reorganization of the actin cytoskeleton, and dysfunctions in synaptic actin dynamics may be closely involved in neurological disorders and dementia [32]. The RhoA/ROCK2/LIMK1/cofilin signalling pathway is a well-known signalling cascade for the regulation of actin dynamics [33, 34]. Previous studies have shown that PirB can regulate the activity of ROCK2 [35]. Our results show that the expression levels of RhoA were significantly increased in CSR mice but not in PirB cKO + CSR mice (Fig. 5a, b), indicating a possible role for PirB in the upregulation of RhoA during CSR. The expression levels of RhoA signalling cascade downstream effectors, Rho-associated coiled-coil containing protein kinase 2 (ROCK2), were significantly increased, and phosphorylation of LIMK1 at threonine 508 (pLIMK1) was significantly reduced in the hippocampi of CSR mice but not in those of PirB cKO + CSR mice (Fig. 5a, c, d). No statistically significant difference was detected in the levels of RhoA, ROCK2, and pLIMK1 between PirB cKO mice and control mice, which might have been due to the ceiling effect (Fig. 5a–d). Cofilin, named actin-depolymerizing factor, is the key downstream effector of LIMK1 and is inactivated by phosphorylation at Ser3 [36]. We also observed that levels of the inactive form of cofilin (pcofilin) were significantly decreased in the hippocampi of CSR mice but not in those of PirB cKO + CSR mice (Fig. 5a, e). Altogether, these data reveal that CSR-mediated activation of the RhoA/ROCK2/LIMK1/cofilin signalling cascade via the PirB receptor may result in abnormal actin dynamics and actin rearrangement, ultimately leading to synaptic deficits and cognitive impairment.

### RhoA/ROCK2 Inhibition Ameliorates Neurological Dysfunction and Synaptic Deficits in CSR Mice

**Fig. 6:**
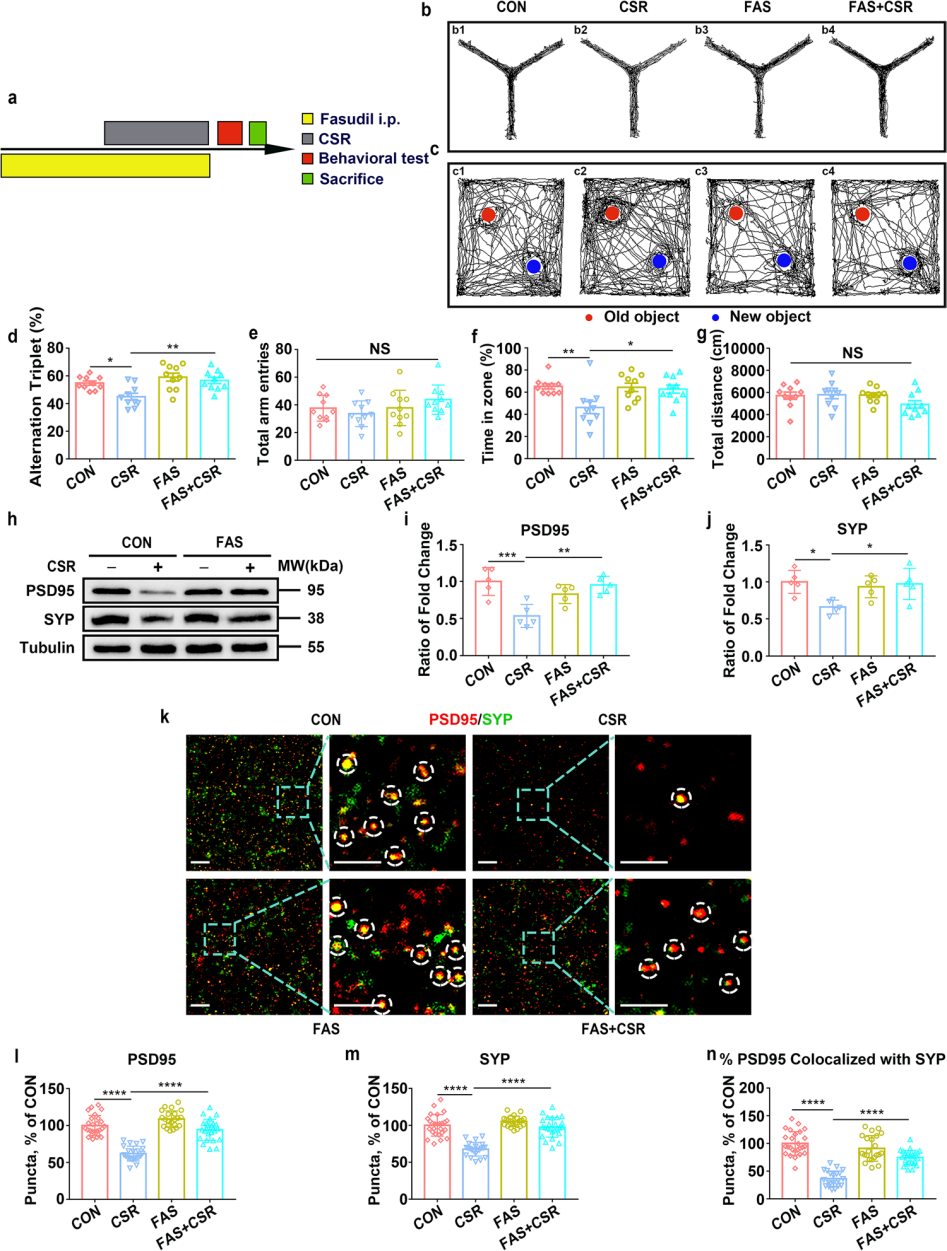
Effect of RhoA/ROCK2 inhibition on cognitive impairment and synapse loss in CSR mice. **a** The experimental protocol for fasudil intraperitoneal injection and CSR is presented. **b**, **c** Representative Y maze and NOR test trajectories of control, CSR, fasudil, and fasudil + CSR mice. **d**, **e** The percent of spontaneous alternation rates and total number of arm entries in the Y maze test were measured (*n* = 10). **f**, **g** The recognition index and total travel distance in the NOR test were measured (*n* = 10). **h** Western blot analysis of hippocampal PSD95 and SYP levels in the four groups of mice. **i**, **j** Ratio of fold change from Western blot. The PSD95 (*n* = 5) and SYP (*n* = 5) levels were significantly higher in fasudil + CSR mice than in CSR mice. **k** Superresolution SIM images of PSD95 (red) and SYP (green) immunoreactive puncta in the hippocampal CA1 region of the four groups of mice. Scale bars: 5 μm. **l**–**n** Quantification of synaptic puncta or their apposition indicates significantly higher PSD95 (*n* = 22–26), SYP (*n* = 22–26), and apposition (*n* = 22–26) levels in the hippocampal CA1 region of fasudil + CSR mice than in CSR mice. Data are presented as the means ± SD and were analysed by one-way ANOVA with Tukey’s test (*p < 0.05, **p < 0.01, ****p < 0.0001)

Finally, we investigated whether RhoA/ROCK2 pathway inhibition using fasudil, a widely used ROCK2 inhibitor, has therapeutic effects on cognitive impairment and synaptic deficits following CSR. First, we verified that intraperitoneal administration of fasudil (10 mg/kg) reversed the upregulation of ROCK2 expression and the downregulation of pLIMK1 and pcofilin expression levels in the hippocampi of CSR mice (Fig. 5f–i). Subsequently, we found that the lower learning and memory capacities in the Y maze (Fig. 6a, b, d, e) and novel object recognition tests (Fig. 6c, f, g) observed in the CSR mice were markedly ameliorated by fasudil (Fig. 6d, f). Next, Western blot analysis showed dramatically higher protein expression levels of both SYP and PSD95 in FAS + CSR mice than in CSR mice (Fig. 6h–j). Moreover, the quantification of colocalized SYP and PSD95 puncta revealed a significant restoration of synapse density in FAS + CSR mice (Fig. 6k–n).

**Fig. 7:**
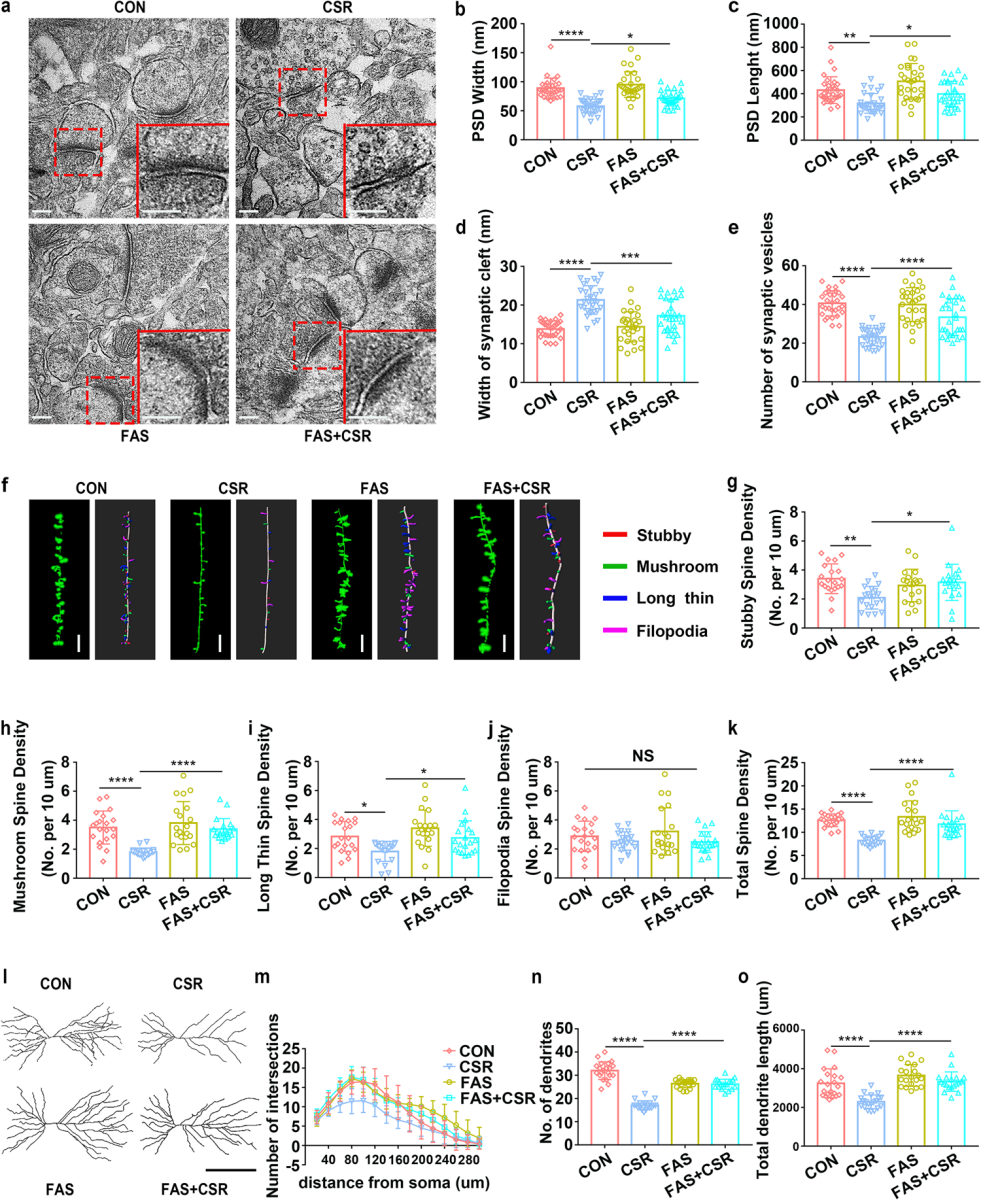
Effect of RhoA/ROCK2 inhibition on synaptic structural deficits in CSR mice. **a** TEM study of ultrastructural features of synapses in the hippocampal CA1 region in control and CSR mice in the presence or absence of fasudil treatment. Scale bars: 200 nm. **b** The width of the PSD (*n* = 29–30). **c** The length of the PSD (*n* = 29–30). **d** The width of the synaptic cleft (*n* = 29–30). **e** The number of synaptic vesicles (*n* = 29–30). **f** Representative confocal and 3D reconstruction images of dendrites of hippocampal CA1 pyramidal neurons obtained from four groups of mice. Scale bars: 5 μm. **g**–**k** Summary of the density of stubby (*n* = 20)-, mushroom (*n* = 20)-, long thin (*n* = 20)-, and filopodia (*n* = 20)-shaped spines and the total spine density (*n* = 20) on dendrites of hippocampal CA1 pyramidal neurons of four groups of mice. **l** Representative images of pyramidal neurons in the hippocampal CA1 region derived from the four groups of mice. Scale bars: 100 μm. **m** Sholl’s analysis of the dendritic branching complexity of the four groups of mice (*n* = 19–21). **n**, **o** Sholl’s analysis of total dendritic length and number of dendrites of four groups of mice (*n* = 19–21). Data from **b** to **k**, **n**, and **o** are presented as the mean ± SD and were analysed by one-way ANOVA with Tukey’s test. Data from **m** are presented as the mean ± SD and were analysed by two-way ANOVA with Tukey’s test (*p < 0.05, **p < 0.01, ***p < 0.001, ****p < 0.0001)

In addition, TEM showed that fasudil significantly reversed the reduction in the number of presynaptic vesicles, postsynaptic PSD length, and PSD width and the increase in synaptic cleft width induced by CSR (Fig. 7a–e). Then, Golgi staining and 3D reconstruction demonstrated that fasudil led to higher proportions of stubby, mushroom, and long thin spines and higher total spine density in FAS + CSR mice than in CSR mice (Fig. 7f–k). Sholl’s analysis showed that the dendritic complexity of CA1 pyramidal neurons was markedly decreased in CSR mice, which was reversed by fasudil (Fig. 7l, m). In particular, significant differences were detected at Sholl’s radii of 40–220 μm in the basal hemisphere between FAS + CSR and CSR mice (Fig. 7m). Moreover, the reduction in total dendritic length and numbers of dendrites in CA1 pyramidal neurons were also significantly reversed by fasudil to levels similar to those of control mice (Fig. 7l, n, o). These findings collectively suggested that intraperitoneal administration of fasudil ameliorates synaptic deficits after CSR by increasing dendritic complexity and synaptic spine density through the RhoA/ROCK2/LIMK1/cofilin signalling pathway, thereby improving cognitive function.

## Discussion

In the current study, the effects of PirB in the hippocampus on the alterations of cognitive function, synapse structure, and the involved molecular pathways activated after CSR were comprehensively investigated. The results led us to propose a model, and a representative schematic diagram is shown in the graphic summary illustration. CSR induces disruption in dendrite structure and synapse loss, resulting in cognitive impairment, accompanied by upregulation of PirB expression levels in the hippocampus. Upregulated levels of PirB can promote increased RhoA levels and in turn cause increased expression levels of ROCK2 and subsequently decreased phosphorylation levels of LIMK1, which further reduces levels of phosphorylated cofilin. Abnormal cofilin activity results in the reorganization of the actin cytoskeleton and disorders in synaptic plasticity, eventually leading to cognitive impairment. PirB knockdown significantly ameliorated synaptic deficits and cognitive dysfunction after CSR through the RhoA/ROCK2/LIMK1/cofilin signalling pathway. Furthermore, administration of the clinically approved ROCK2 inhibitor fasudil alleviated the disruption in dendrite structure, synapse loss, and impaired cognitive function induced by CSR. Thus, this research primarily clarifies the role and mechanism of PirB in cognitive impairment and offers translational evidence for PirB as a potential therapeutic target for cognitive impairment following CSR.

The most striking finding of this research was the identification of PirB as a key determinant of cognitive impairment after CSR. Sleep and sleep loss affect important brain functions, such as alertness, cognitive function, and mood, by regulating synaptic dynamics and synaptic efficacy [37–39]. A recent study showed that a brief period of 5 h of acute sleep deprivation leads to cognitive impairments in young adult mice [11]. Although it is widely believed that CSR impairs hippocampal cognitive function, the mechanisms remain unknown. Our work reveals that CSR induces cognitive impairment and synaptic deficits and increases PirB expression levels in the hippocampus. To further test the causal relationship between expression changes of PirB and cognitive impairment, we knocked down PirB and found that PirB deficiency reversed cognitive dysfunction after CSR. Previous studies have confirmed that PirB expression level in neurons is significantly increased after neurological injuries, including encephalitis [40], brain aging [41], and stroke [14]. The blockade of PirB activity or the knockdown of PirB expression by antibody antagonism or genetic approaches significantly improved short- and long-term cognitive function in mice after neurological injuries, even in normal brains [21]. Despite the application of different disease models, our findings in CSR are consistent with those in previous studies. In addition, studies have shown that fasudil treatment is able to improve cognitive dysfunction in stroke and Alzheimer’s disease by inhibiting the hippocampal ROCK/cofilin pathway [42, 43], and these pieces of evidence provide strong support for our findings that fasudil can ameliorate cognitive dysfunction after CSR. In all, these results suggest that PirB in the hippocampus negatively regulates cognitive function after CSR.

How PirB in the hippocampus then modulates the consequences of cognitive impairment after CSR remains unknown. It has been reported that PirB is involved in the regulation of synaptic plasticity and related cognitive functions under physiological conditions and in a variety of disease models. Deletion of PirB in the visual cortex and hippocampal neurons results in a significant increase in dendritic spine density and synaptic plasticity [19–22, 44]. In addition, acute blockade of PirB function in the primary motor cortex L5PNs of adult WT mice increases the density and survival of learning-induced spines and enhances motor learning [18]. In agreement with the results of previous studies, our data show that PirB deficiency results in significant improvement of synaptic deficits in hippocampal CA1 pyramidal neurons after CSR. It is well accepted that functional plasticity is closely related to structural plasticity, and both are involved in many pathophysiological processes, such as acute sleep deprivation [36]. Consistent with the above research, our electrophysiological results also confirmed that the knockdown of PirB reversed the reduction in synaptic transmission induced by CSR. Overall, these results suggest that the knockdown of PirB ameliorates CSR-related cognitive impairment by improving structural and functional synaptic deficits in hippocampal CA1 neurons.

However, the exact mechanism by which PirB regulates the structural plasticity and synaptic transmission of hippocampal pyramidal neurons and further modulates cognitive impairment after CSR is completely unknown thus far. The RhoA/ROCK2/LIMK1/cofilin pathway is a well-known signalling cascade that regulates actin dynamics, including during acute sleep deprivation [28, 33, 36]. Rearrangement of the actin cytoskeleton is involved in the regulation of the morphology and function of synaptic spines and hence plays an important role in the pathophysiological process of various diseases [45, 46], such as the regulation of axon regeneration after central nervous system injury [47]. Moreover, PirB can regulate the activity of ROCK2 [35], and RhoA plays an important role in the synaptic plasticity and morphogenesis of dendritic spines of neurons by regulating the organization of the actin cytoskeleton [48]. Further studies showed that activated RhoA triggered cytoskeletal recombination by activating ROCK2 and downstream effectors [49]. In line with the results of previous studies, our results reveal that the expression levels of RhoA in the hippocampus were significantly increased and that levels of the RhoA effector ROCK2 were also increased after CSR. The downstream target of RhoA/ROCK2 activation is LIMK1, the activation of which is involved in the regulation of growth cone collapse and neurite outgrowth inhibition [50, 51]. In turn, LIMK1 phosphorylated at the Ser3 residue possesses the ability to inhibit the actin-depolymerizing protein cofilin by phosphorylation [52, 53], and changes in cofilin activity are essential for the reorganization of the actin cytoskeleton [54]. We further observed that CSR decreases the phosphorylation levels of LIMK1 and cofilin in the hippocampus. In support of our observation, acute sleep deprivation reduces the phosphorylation levels of LIMK1 and cofilin in the hippocampus [11, 36]. Surprisingly, knockdown of PirB partially reversed the modulation of RhoA/ROCK2/LIMK1/cofilin signalling pathways after CSR. To further confirm that RhoA/ROCK2/LIMK1/cofilin signalling pathways are involved in the regulation of the effect of PirB on cognitive function, structural plasticity, and synaptic transmission after CSR, fasudil, a clinically approved ROCK2 inhibitor, was selected. Notably, fasudil partially rescued the synaptic deficits and cognitive impairment caused by CSR. Previous studies have demonstrated that administration of the ROCK2 inhibitor fasudil could prevent the reduction in dendritic arbour complexity and loss of dendritic spines in Alzheimer’s disease mice [55], which further consolidated our findings. Collectively, the results of our study reveal that PirB regulates the actin cytoskeleton through the RhoA/ROCK2/LIMK1/cofilin signalling pathway, which participates in the regulation of structural plasticity and synaptic transmission as well as cognitive function following CSR.

Our findings are inconsistent with the synaptic homeostasis hypothesis, which holds that wakefulness leads to a net increase in synaptic strength, while sleep results in a decrease in synaptic strength and synaptic spine density [56–58]. In recent years, numerous studies have provided data that support the synaptic homeostasis hypothesis. In adolescent mice, it was found that waking leads to a net gain in cortical spines, and sleep results in net spine loss in the somatosensory cortex [59]. Beyond the changes in structural plasticity, wakefulness also seems to be associated with net synaptic potentiation, while sleep may benefit global synaptic inhibition in the cortex and hippocampus of rats, which is also in line with synaptic homeostasis theory [60]. However, there is also a great deal of data that do not agree with the synaptic homeostasis hypothesis. In mice, 5 h of acute sleep deprivation decreases the numbers of hippocampal CA1 dendritic spines, and recovery sleep normalizes the alterations of this structure [11]. Consistent with these findings, we detected a dramatic increase in dendrite structure disruption and synapse loss and a reduction in synaptic transmission after CSR. Sleep does not have a single effect on synaptic strength and efficacy, and such differences in effect may be found across different brain regions, ages of the subjects, and species.

In addition to being expressed in neurons, double-labelled immunofluorescence staining confirmed that PirB was also slightly expressed in astrocytes but not microglia in the central nervous system [15, 40]. To focus our research, the effects of PirB receptors on astrocytes in cognitive impairment and synaptic deficits due to CSR were not investigated, and the possible implications of the PirB receptors on astrocytes in the hippocampus in mediating synaptic plasticity and memory changes associated with CSR remain to be explored further in the future.

In conclusion, this research elucidates that PirB participates in the regulation of hippocampal-dependent structural and functional plasticity and cognitive function after CSR by activating the RhoA/ROCK2/LIMK1/cofilin signalling pathway. Furthermore, we identified fasudil as a potential therapeutic candidate for the treatment of cognitive impairment induced by CSR. In the further, targeting PirB might present an attractive strategy to counteract cognitive impairment after CSR.

## Data Availability

The datasets used and/or analysed during the current study are available from the corresponding author on reasonable request.

## Abbreviations

PirB: Paired immunoglobulin-like receptor B
CSR: Chronic sleep restriction
SD: Sleep deprivation
LILRB2: Leukocyte immunoglobulin (Ig)-like receptor B2
ITIMs: Immunoreceptor tyrosine-based inhibitory motifs
PirB cKO: PirB conditional knockdown
NOR: Novel object recognition test
AAV: Adeno-associated virus
PFA: Paraformaldehyde
TEM: Transmission electron microscopy
FAS: Fasudil
SYP: Synaptophysin
PSD95: Postsynaptic density protein

## Code Availability

GraphPad Prism Version 7.0; ImageJ Version 1.51 J; Imaris Version 9.7.
